# A flow cytometry-based analysis to establish a cell cycle synchronization protocol for *Saccharum* spp.

**DOI:** 10.1038/s41598-020-62086-9

**Published:** 2020-03-19

**Authors:** Shan Yang, Kai Zeng, Ling Luo, Wang Qian, Zhiqiang Wang, Jaroslav Doležel, Muqing Zhang, Xiangxiong Gao, Zuhu Deng

**Affiliations:** 1 National Engineering Research Center for Sugarcane, Fujian Agriculture and Forestry University, Fuzhou, 350002 China; 2 State Key Laboratory for Protection and Utilization of Subtropical Agro-Bioresources, Guangxi University, Nanning, 530004 China; 3 Centre of Plant Structural and Functional Genomics, Institute of Experimental Botany, Olomouc, CZ-78371 Czech Republic; 4 College of Horticulture, Fujian Agriculture and Forestry University, Fuzhou, 350002 China

**Keywords:** Cell biology, Plant sciences

## Abstract

Modern sugarcane is an unusually complex heteroploid crop, and its genome comprises two or three subgenomes. To reduce the complexity of sugarcane genome research, the ploidy level and number of chromosomes can be reduced using flow chromosome sorting. However, a cell cycle synchronization (CCS) protocol for *Saccharum* spp. is needed that maximizes the accumulation of metaphase chromosomes. For flow cytometry analysis in this study, we optimized the lysis buffer, hydroxyurea(HU) concentration, HU treatment time and recovery time for sugarcane. We determined the mitotic index by microscopic observation and calculation. We found that WPB buffer was superior to other buffers for preparation of sugarcane nuclei suspensions. The optimal HU treatment was 2 mM for 18 h at 25 °C, 28 °C and 30 °C. Higher recovery treatment temperatures were associated with shorter recovery times (3.5 h, 2.5 h and 1.5 h at 25 °C, 28 °C and 30 °C, respectively). The optimal conditions for treatment with the inhibitor of microtubule polymerization, amiprophos-methyl (APM), were 2.5 μM for 3 h at 25 °C, 28 °C and 30 °C. Meanwhile, preliminary screening of CCS protocols for Badila were used for some main species of genus *Saccharum* at 25 °C, 28 °C and 30 °C, which showed that the average mitotic index decreased from 25 °C to 30 °C. The optimal sugarcane CCS protocol that yielded a mitotic index of >50% in sugarcane root tips was: 2 mM HU for 18 h, 0.1 X Hoagland’s Solution without HU for 3.5 h, and 2.5 μM APM for 3.0 h at 25 °C. The CCS protocol defined in this study should accelerate the development of genomic research and cytobiology research in sugarcane.

## Introduction

Sugarcane (*Saccharum* spp.), belonging to the genus *Saccharum*, is an economically valuable crop. The genus *Saccharum* is diverse in genome content and organization and comprises two wild species (*S. robustum* and *S. spontaneum*) and four groups of former cultivated clones (*S. officinarum*, *S. sinense*, *S. bareri* and *S. edule*)^1^. *S. officinarum* is thought to have evolved from *S. robustum*^2^.

The genome of modern sugarcane contains 100–130 chromosomes, of which 80–90% are from *S. officinarum* and 10–20% are from *S. spontaneum*^3^. Thus, sugarcane has an unusually complex, highly polyploid and aneuploid genome that complicates analyses of genome sequence and assembly. Although genome sequencing on the tetraploid *S. spontaneum* has been performed, the assembly accuracy was not high and many gene sequences were absent^4^. To reduce this complexity, the sugarcane genome can be dissected into single chromosomes using flow cytometry. Since this approach requires sufficient numbers of metaphase chromosomes, a stable and efficient cell cycle synchronization (CCS) method for sugarcane is needed.

The eukaryotic cell cycle is typically divided into four phases: G_1_ phase, in which the cell grows and duplicates organelles; S phase, in which DNA synthesis occurs; G_2_ phase, in which the cell prepares to divide after replication; M phase, during which the chromosomes precisely separate and form two daughter nuclei along the mitotic spindle, and cytokinesis when the actual cell division occurs^5–7^. Cells in each of these cell cycle phases have a distinct nuclear DNA content, which can be exploited for sorting by flow cytometry^8^. Furthermore, cell synchronization followed by flow cytometry can be used to enrich large populations of cells in a given phase^9,10^. For plants in particular, isolation of metaphase chromosomes is essential for cytogenetic, cytobiologic and genomic studies^11–13^, yet cells in this phase represent only 5–10% of the total cell population. Moreover, cell cycle progression in plant tissues proceeds asynchronously^14^.

Artificial CCS is a prerequisite for obtaining high concentrations of chromosomes. Stable synchronization schemes for various plants have been established using actively growing cell cultures or root tips^15,16^. In contrast to cell culture, plant root tips are a good source of chromosomes and are also cheaper, more stable and easier to handle^17^. As such, increasing numbers of researchers are using plant root tips as starting materials for CCS studies. To date, there are two main CCS strategies: (i) collection of specific cell populations using physical methods such as centrifugal elutriation^9^; and (ii) treatment of cells with chemical inhibitors that impede DNA synthesis inhibitors or microtubule polymerization as well as other metaphase blocking chemicals^15^. CCS in root tips from several crops, including cereal, wheat, Chinese fir and *Vicia faba* can be achieved with chemical inhibitors ^17–20^. Moreover, DNA synthesis inhibitors such as hydroxyurea (HU), deoxyadenosine and deoxythymidine, and microtubule inhibitors such as trifluralin and amiprophos-methyl (APM), are effective for CCS in plant root tips^8^. DNA synthesis inhibitors and microtubule inhibitors together can also be more effective to achieve CCS than either agent alone^13,18^.

The timing of the mitotic cycle of different plant species varies according to genome size. Therefore, given the complexity of the sugarcane genome and the variety of clones, developing a stable CCS method has been challenging. Based on results obtained for predecessors in other crops, in this study we examined the effect of treatment temperature, chemical inhibitor concentration and processing time. We analyzed different chemical inhibitor concentrations and processing times for Badila (*S. officinarum*, 2n = 8 × =80) roots at different temperatures by flow cytometry and microscopy to compile preliminary CCS protocols, which we then screened using different temperatures and different *Saccharum* clones. Finally, we defined a stable and optimal CCS protocol for sugarcane, which could greatly promote the development of sugarcane cytology and genomics.

## Material and methods

### Plant material

In this study, 5 *S. officinarum* clones, 8 *S. spontaneum* clones, 5 *S. robustum* clones, 1 *Saccharum sinense* clone, 2 *S. barberi* clones *a*nd 7 *Saccharum* hybrid clones were used for CCS (Table 1). All clones were obtained from the Fujian Agricultural and Forestry University sugarcane germplasm resources nursery (Fuzhou, China) and were cut into single bud stems. These single bud stems were cleaned, soaked in 0.5% carbendazim solution for 24 h, placed in a pallet and covered with perlite, kept moist with ddH_2_O and incubated in the dark at 25 ± 0.5 °C, 28 ± 0.5 °C or 30 ± 0.5 °C in a biological incubator.

**Table 1:** *Saccharum* species used in this study.

No.	Species	Name
1	*Saccharum officinarum*	14NG124
2	*Saccharum officinarum*	51NG-103
3	*Saccharum officinarum*	Badila
4	*Saccharum officinarum*	Guan A
5	*Saccharum officinarum*	LA Purple
6	*Saccharum spontaneum*	12–165
7	*Saccharum spontaneum*	2007–11
8	*Saccharum spontaneum*	87–20
9	*Saccharum spontaneum*	Laos-2
10	*Saccharum spontaneum*	SES-208
11	*Saccharum spontaneum*	82–63
12	*Saccharum spontaneum*	82–110
13	*Saccharum spontaneum*	82–114
14	*Saccharum robustum*	51NG3
15	*Saccharum robustum*	51NG63
16	*Saccharum robustum*	51NG155
17	*Saccharum robustum*	NG77–004
18	*Saccharum robustum*	57NG208
19	*Saccharum sinense*	Songxizhe
20	*Saccharum barberi*	Nagans
21	*Saccharum barberi*	Pansahi
22	*Saccharum* hybrid	ROC22
23	*Saccharum* hybrid	Guitang30
24	*Saccharum* hybrid	Mintang01–77
25	*Saccharum* hybrid	Yuetang96–86
26	*Saccharum* hybrid	Yunzhe0551
27	*Saccharum* hybrid	ROC10
28	*Saccharum* hybrid	Funong40

### Selection of suitable lysis buffer for nuclei suspension

Badila roots without CCS were cut to a 1.0 cm length, rinsed in ddH_2_O, and fixed in a 2% formaldehyde fixative solution for 20 min at 4 °C. The roots were then washed three times with Tris buffer with 5 min/wash. A total of 30 root tips, 1.0–1.5 mm in length, were cut off from the intact stems using a sterile scalpel in a glass Petri dish and then collected into a 1.5 mL tube containing 1 ml of the indicated lysis buffer (Table 2). Nuclei were isolated from the root tips using a Polytron PT1300 homogenizer (Kinematica AG, Litau, Switzerland) at 9,600 rpm for 18 s, and the nuclei suspension was filtered through a 60 µm nylon mesh. The quality of the nuclei suspensions was evaluated after flow cytometry.

**Table 2:** Buffers used in this study.

Buffer	Composition	References
LB01	15 mM Tris; 2 mM Na_2_EDTA·2H_2_O; 0.5 mM spermine·4HCl; 80 mM KCl; 20 mM NaCl; 15 mM HOCH_2_CH_2_SH; 0.1% (v/v) Triton X-100; pH 7.5	^15^
MgSO_4_	9.53 mM MgSO_4_·7H_2_O; 47.67 mM KCl; 4.77 mM HEPES; 6.48 mM DTT; 0.25% (v/v) Triton X-100; pH 8.0	^36^
WPB	0.2 M Tris·HCl; 4 mM MgCl_2_·6H_2_O; 2 mM Na_2_EDTA·2H_2_O; 86 mM NaCl; 10 mM Na_2_S_2_O_5_; 1% PVP-10; 1%(v/v) Triton X-100; pH 7.5	^23^
GPB	0.5 mM spermine·4HCl; 30 mM Na_3_C_6_H_5_O_7_·2H_2_O; 20 mM MOPS; 80 mM KCl; 20 mM NaCl; 0.5% (v/v) Triton X-100; pH 7.0	^23^
Bino’s	200 mM mannitol; 10 mM MOPS; 0.05% (v/v) Triton X-100; 10 mM KCl; 10 mM NaCl; 2.5 mM DTT; 10 mM spermine·4HCl; 2.5 mM Na_2_EDTA·2H_2_O; 0.05% (w/v) sodium azide; pH 5.8	^37^
Otto’s	Otto I: 100 mM C_6_H_8_O_7_·H_2_O; 0.5% (v/v) Tween 20; pH approx. 2–3 Otto II: 400 mM Na_2_PO_4_·12H_2_O; pH approx. 8–9	^38^
Formaldehyde fixative	10 mM Tris; 10 mM Na_2_EDTA; 100 mM NaCl; 0.1% Triton X-100; 2% formaldehyde stock solution(37%, v/v); pH 7.5	^16^
Tris buffer	10 mM Tris; 10 mM Na_2_EDTA; 100 mM NaCl; pH 9	^15^

### Preparation of nuclei suspensions

WPB buffer was used to prepare nuclei suspensions. The roots from different treatments were cut into 1.0 cm sections and subjected to the same steps as described above. All suspensions were stored at 4 °C until use.

### Cell cycle synchronization

CCS was carried out according to the method described by Vrana *et al*.^21^ using the following steps:

#### DNA synthesis inhibition

The roots were washed in ddH_2_O until a 1.5 cm length was achieved. The roots were then transferred to a 200 mL plastic tray containing 100 mL 0.1X Hoagland’s Solution (HS) containing hydroxyurea (HU) at 0.625, 1.25, 2, 2.5, 3, 3.5 or 5.0 mM, immersed in the solution and incubated in the dark at the indicated temperature (25 °C, 28 °C and 30 °C). Meanwhile, oxygen was added to the solution during incubation using an oxygen pump. Root tips (1.0 cm long) from five stems were cut off at 2 h intervals across a 26 hour period (0–26 h) after incubation in solutions having varying amounts of HU. The obtained roots were used to prepare nuclei suspensions. The optimal processing time and HU concentration was determined by flow cytometry.

#### Recovery treatment

The roots were thoroughly rinsed in ddH_2_O and transferred to 0.1X HU-free HS after optimal HU treatment. Oxygen was infused into the solution throughout the entire recovery period using an oxygen pump. Roots were cut off at 30 min intervals between 0 h and 6 h and used to prepare nuclear suspensions and to determine the optimal recovery time by flow cytometry.

#### Microtubule inhibition

To enrich mitotic cells, roots were immersed in solutions containing 0.625, 1.25, 2.5, 3.5 or 5.0 µM of amiprophos-methyl (APM). Root tips were cut off at 1.0 h intervals between 0 h and 7 h after optimal recovery treatment. The resulting roots were used to determine the metaphase index using microscopy.

### Mitotic index analysis

For each treatment condition, 1.0 cm long sections from 20 roots were isolated, washed in ddH_2_O and fixed overnight in ethanol:glacial acetic acid (v: v = 3:1) at 4 °C before successively rinsing for 10 min with 100% ethanol, 95% ethanol and 75% ethanol. The treated root sections were stored at −20 °C in 70% ethanol. The root-tips were cut into 1.0–1.5 mm sections and transferred to enzyme mixtures containing 4% cellulose (Sigma), 1% pectolyase (Sigma), and 0.5% pectinase (Sigma) and incubated at 37 °C for 1 h. The root tips were then washed in ddH_2_O, hydrolyzed in 5 M HCl for 20 min at room temperature, washed three times in ddH_2_O, and stained with Schiff’s reagent (pararosaniline; Sigma-Aldrich) for 1 h at room temperature. To prepare slides for observation of cells, 10 uniform root tips were selected. More than 1,000 cells per slide were randomly selected and photographed using an AxioCam MRc5 and AxioVision v. 4.7 software (Carl Zeiss Microscope, Gottingen, Germany). The average mitotic index was estimated based on 30 images with at least 3000 cells. The mitotic index data were analyzed and plotted using Origin 9.1.

$${\rm{Mitotic}}\,{\rm{index}}=\frac{{\rm{Total}}\,{\rm{number}}\,{\rm{of}}\,{\rm{mitotic}}\,{\rm{cells}}\,{\rm{per}}\,{\rm{image}}}{{\rm{Total}}\,{\rm{number}}\,{\rm{of}}\,{\rm{cells}}\,{\rm{per}}\,{\rm{image}}}$$

### Flow cytometry

DAPI solution (20 μL, 0.1 mg/mL) was added to 1.0 mL nuclei suspensions. After DAPI staining, nuclei suspensions from different samples were analyzed using a Becton-Dickinson Influx flow cytometer (Guava easyCyte 12HT, USA) at a 300–500/s flow rate and excitation at 355 nm. In regard to flow cytometer, the basic CV value of FSC, SSC and DAPI are 0.39% 1.19%and 1.03%, respectively. Data were collected in 10,000 particle units and used to form a univariate flow karyotype histogram of fluorescence area signals and analyzed by FlowJo software to show the percentage of nuclei in the G_1_, S, and G_2_/M phases.

### Statistical analysis

A completely randomized design was used with 3 replications for each treatment. The variance significance analysis among different treatments was tested by one-way ANOVA at 95%, 99% or 99.9% confidence level, and post hoc comparisons were made using the multi-domains Duncan test (*p* < 0.05, *p* < 0.01 or *p* < 0.001). All procedures of statistical analysis were executed by IBM SPSS Statistics 19.0.

## Results

### Selection of optimal lysis buffer to prepare nuclei suspensions

**Figure 1:**
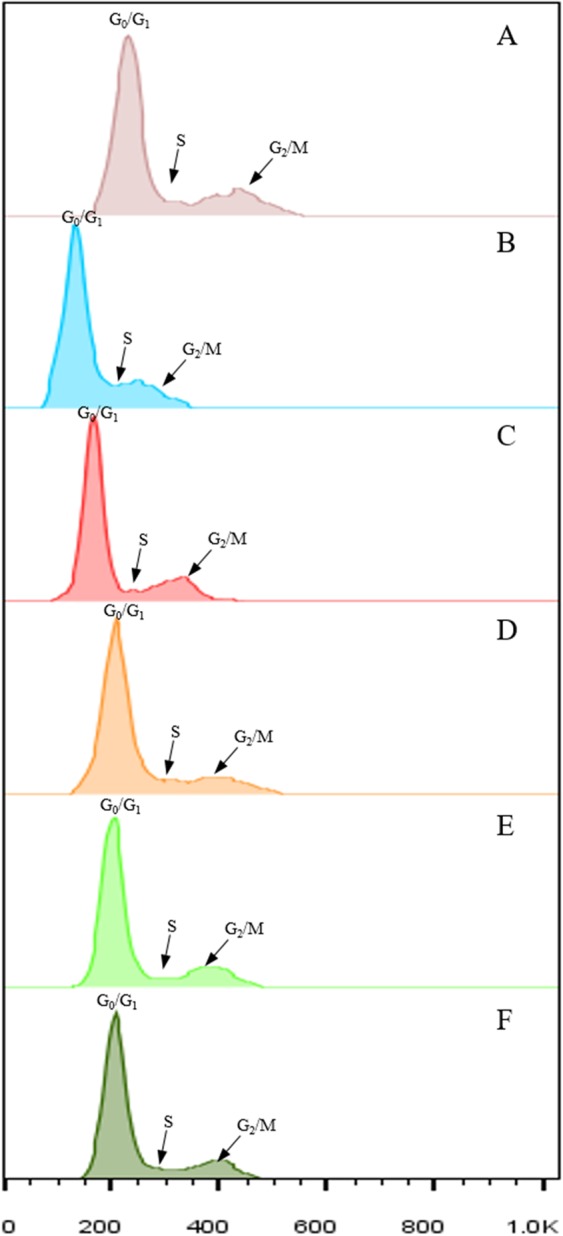
Suspension of sugarcane nuclei prepared using different lysis buffers for cell cycle analysis. (**A**) LB01 buffer. (**B**) MgSO_4_ buffer. (**C**) WPB buffer. (**D**) GPB buffer. (**E**) Bino’s buffer. (**F**) Otto’s buffer. Three independent biological replicates with at least 10,000 nuclei per sample.

The peak of the G_2_/M phase in flow cytometry was flattened using MgSO_4_ buffer, GPB buffer, Bino buffer and Otto’s buffer, and thus the S phase could not be distinguished and the proportion of S and G_2_/M phase could not be calculated accurately (Fig. 1). However, nuclei suspended in LB01 or WPB buffers did allow the G_0_/G_1_, S and G_2_/M phases to be distinguished. The G_2_/M peak was broader for suspensions prepared with LB01 buffer relative to those prepared with WPB buffer. The error value of the data for LB01 buffer was larger than that for WPB buffer. Thus, WPB buffer was the most suitable for preparing sugarcane nuclei suspensions.

### Inhibition of DNA synthesis

**Figure 2:**
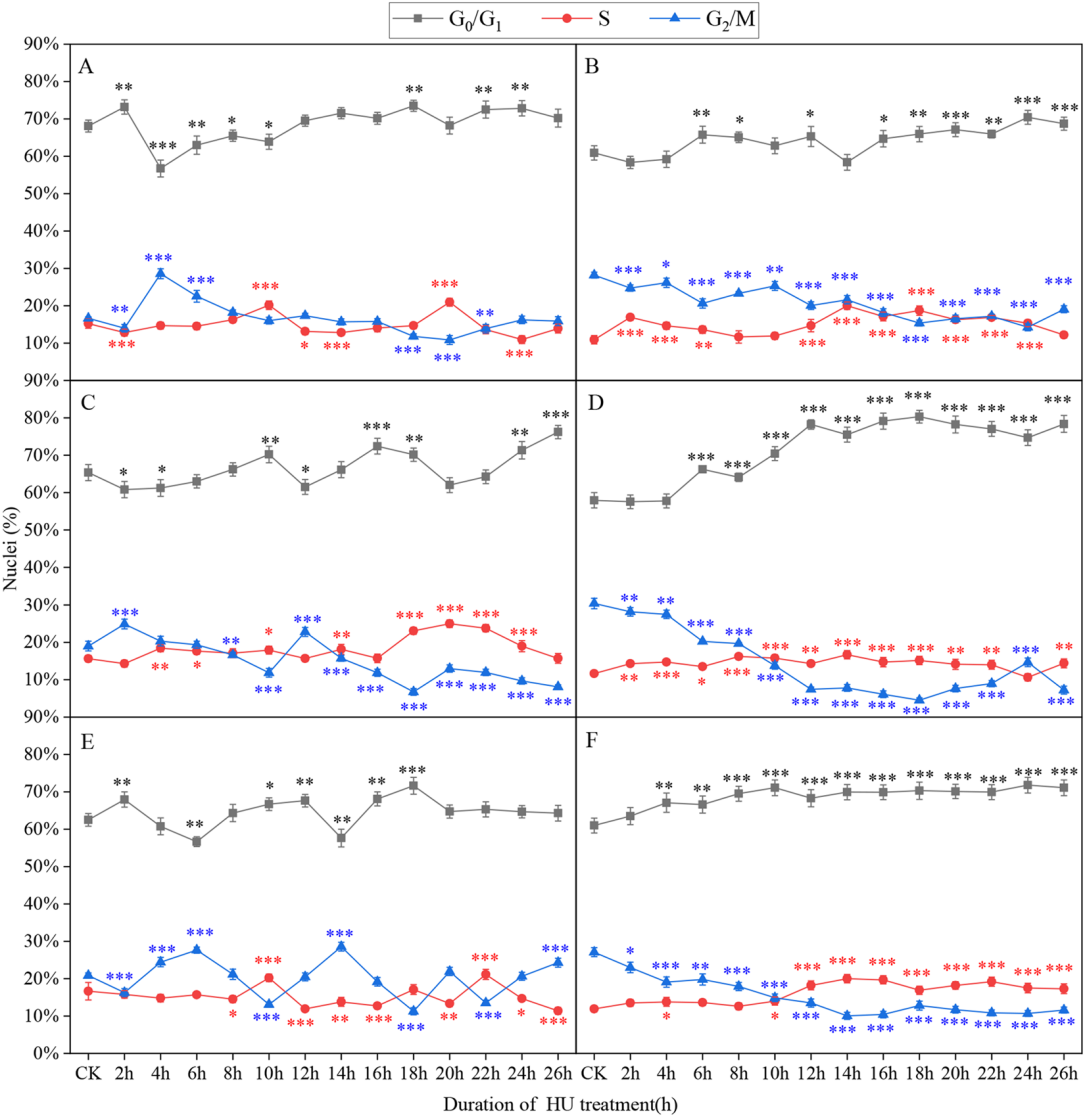
Proportion of cells in different phases after different treatment times and HU concentrations at 25 °C. (**A**) 0.625 mM HU; (**B**) 1.25 mM HU; (**C**) 2 mM HU; (**D**) 2.5 mM HU; (**E**) 3 mM HU; (**F**) 5 mM HU. Three independent biological replicates with at least 10,000 nuclei per sample. The asterisks denote that it is on the level of significance in comparison to CK (*stand for *p* < 0.05, **stand for *p* < 0.01 and ***stand for *p* < 0.001).

Hydroxyurea (HU) treatment could inhibit DNA synthesis and resulted in the accumulation of a large fraction of cells in the G_1_/S phase (Supplementary Dataset Fig. S1). Therefore, we focused on what HU concentration and processing time promoted accumulation of the most cells in the G_0_/G_1_ and S phase. The average percentage of G_0_/G_1_, S, and G_2_/M phase cells was 70.3%, 10.5% and 19.2%, respectively, without HU treatment at 25 °C (Fig. S1-A). HU concentrations below 2 mM had little effect on the accumulation of G_1_/S phase cells between 0 h and 26 h after treatment (Fig. 2A,B). When the HU concentration was increased to 2–5 mM, cells in the G_1_/S phase did accumulate (Fig. 2C–F). However, at HU concentrations above 3 mM, DNA synthesis was completely inhibited and did not recover (Fig. 2E,F). The largest proportion of cells in the G_1_/S phase cells in the root tip cells was seen at 18 h after treatment with 2 mM or 2.5 mM HU, which had similar effectiveness (93.1% and 95.4%, respectively). The time for entry into the G_1_ phase was extended and transition from the S phase to the G_2_ phase was impeded following treatment with 2.5 mM HU. Therefore, 2.0 mM HU treatment was better than others at 25 °C. Meanwhile, we eliminated from consideration treatment with 0.625 mM and 5.0 mM HU at 28 °C or 30 °C, as the percentages of cells in G_1_/S phases were either too low or too high, respectively. So, 1.5 mM, 2.0 mM, 2.5 mM or 3.0 mM of HU was selected at 28 °C and 30 °C.

**Figure 3:**
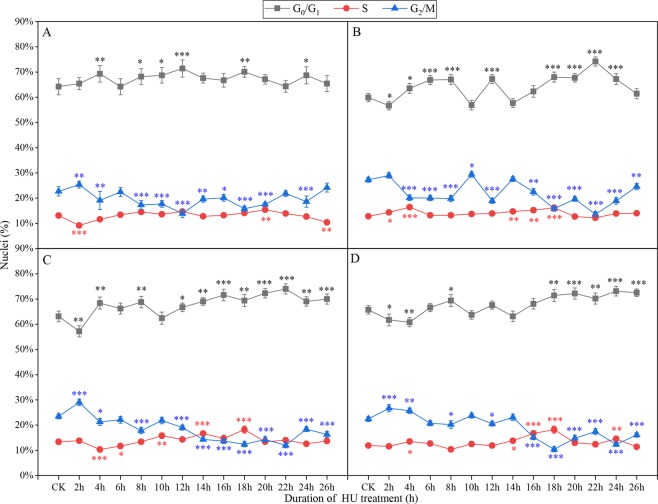
Proportion of cells in different phases after different treatment times and HU concentrations at 28 °C. (**A**) 1.5 mM HU; (**B**) 2 mM HU; (**C**) 2.5 mM HU; (**D**) 3 mM HU. Three independent biological replicates with at least 10,000 nuclei per sample. The asterisks denote that it is on the level of significance in comparison to CK (*stand for *p* < 0.05, **stand for *p* < 0.01 and ***stand for *p* < 0.001).

When Badila roots were incubated in 2 mM HU solution for 18 h or 22 h at 28 °C, there were two peaks for G_1_/S phase cells, which represented 87.2% and 88.4%, respectively, of the total cell population (Fig. 3B). The percentage of G_1_/S phase cells was 87.6% and 88% when roots were incubated in 2.5 mM HU solution for 18 h and 22 h, respectively (Fig. 3C). The largest proportion of G_1_/S phase cells, 89.6%, was seen when roots were incubated in 3.0 mM HU solution for 18 h (Fig. 3D). However, incubation with 2.5 mM and 3.0 mM HU had negative impacts on the cell cycle and recovery was not observed.

**Figure 4:**
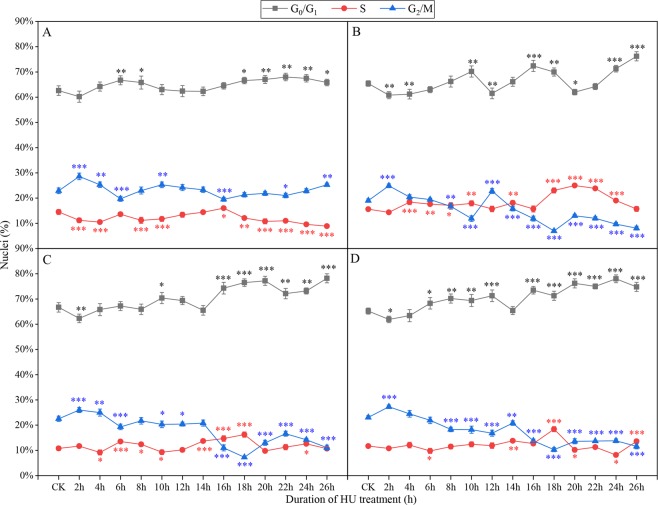
Proportion of cells in different phases after different treatment times and HU concentrations at 30 °C. (**A**) 1.5 mM, (**B**) 2 mM, (**C**) 2.5 mM, (**D**) 3 mM. Three independent biological replicates with at least 10,000 nuclei per sample. The asterisks denote that it is on the level of significance in comparison to CK in the same phase (*stand for *p* < 0.05, **stand for *p* < 0.01 and ***stand for *p* < 0.001).

At 30 °C, the maximum proportion of G_1_/S phase cells was 80.5% when roots were incubated in 1.25 mM HU solution for 17 h (Fig. 4A). After incubation of roots in 2.0 mM, 2.5 mM or 3.0 mM HU solution for 18 h, the proportion of G_1_/S phase cells peaked, with the highest percentage, 93.1%, seen for 2.0 mM HU solution (Fig. 4B–D).

Overall, incubation of root tips at 25 °C, 28 °C or 30 °C promoted accumulation of similar numbers of G_1_/S phase cells, and for Badila roots incubation in 2.0 mM HU solution for 18 h was optimal (Supplementary Dataset Fig. S2).

### Recovery processing

**Figure 5:**
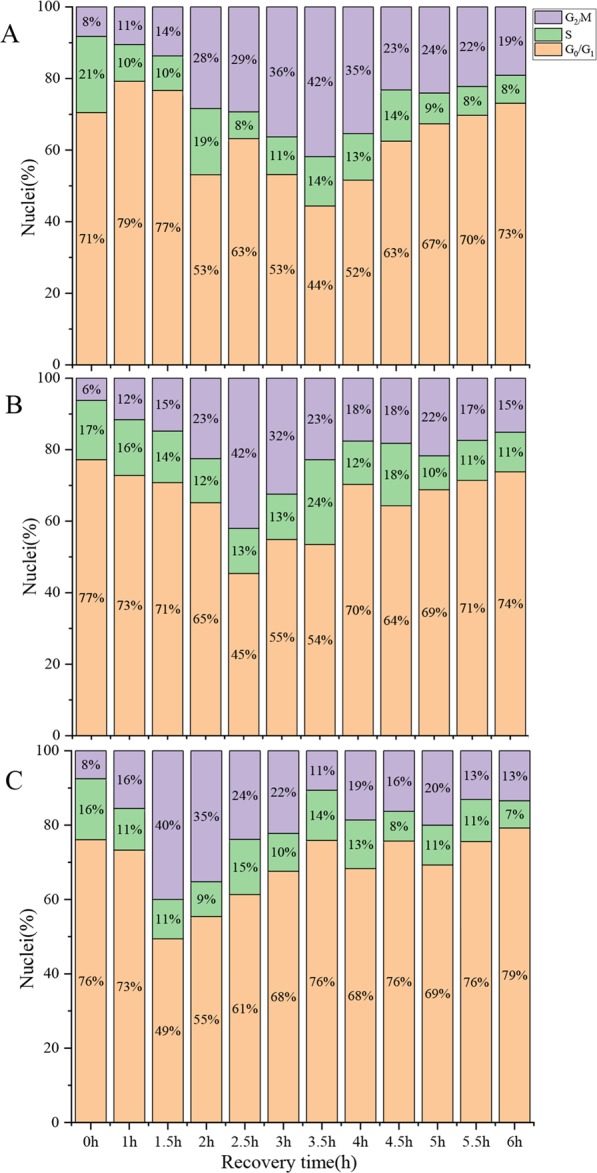
Proportion of cells in different phases after different recovery time treatments at (**A**) 25 °C, (**B**) 28 °C or (**C**) 30 °C. Three independent biological replicates with at least 10,000 nuclei per sample.

In the recovery stage, we needed to know what incubation time in Hoagland’s solution (HS) lacking HU would yield the highest proportion cells in the S and G_2_/M phase. Flow cytometry showed that the percentage of cells in the S and G_2_/M phase peaked at 56% after incubation in HS lacking HU for 3.5 h at 25 °C (Fig. 5). At 28 °C and 30 °C, the maximum proportion of S and G_2_/M phase cells was 55% and 51% respectively, after a 2.5 h and 1.5 h HU-free treatment, respectively (Fig. 5). From these results, we determined that incubation in HU-free HS for 3.5 h, 2.5 h and 1.5 h after 2.0 mM HU treatment for 18 h at 25 °C, 28 °C and 30 °C, respectively, produced optimal amounts of cells in the S and G_2_/M phase.

### Microtubule inhibition

To enrich mitotic cells, Badila roots were treated with APM solution of varying concentrations for 3.5 h, 2.5 and 1.5 h after recovery at 25 °C, 28 °C and 30 °C, respectively.

**Figure 6:**
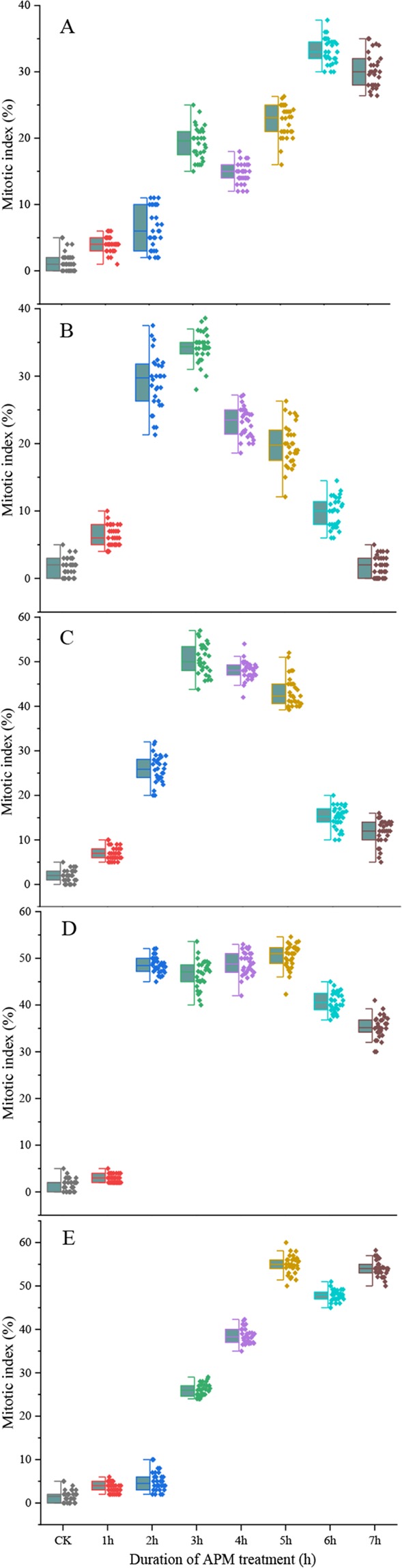
Mitotic index following treatment with different APM concentrations at 25 °C. (**A**) 0.625 μM, (**B**) 1.25 μM, (**C**) 2.5 μM, (**D**) 3.5 μM, E: 5 μM. Thirty independent images with at least 3,000 nuclei per sample.

At 25 °C, the highest percentage of mitotic cells (M phase cells) was approximately 33.2%, 34.3%, 50.5%, 50.4% and 54.9% with 0.625 μM, 1.25 μM, 2.5 μM, 3.5 μM and 5.0 μM APM treatment for 6 h, 3 h, 3 h, 5 h and 5 h, respectively, indicating that an APM concentration of at least 2.5 μM was needed (Fig. 6). Although exposure to higher amounts of APM solution for longer periods produced more mitotic cells, the morphology of these cells was abnormal (data not shown). Therefore, it was optimal to incubate roots in the 2.5 μM APM solution for 3 h at 25 °C for accumulating mitotic cells.

**Figure 7:**
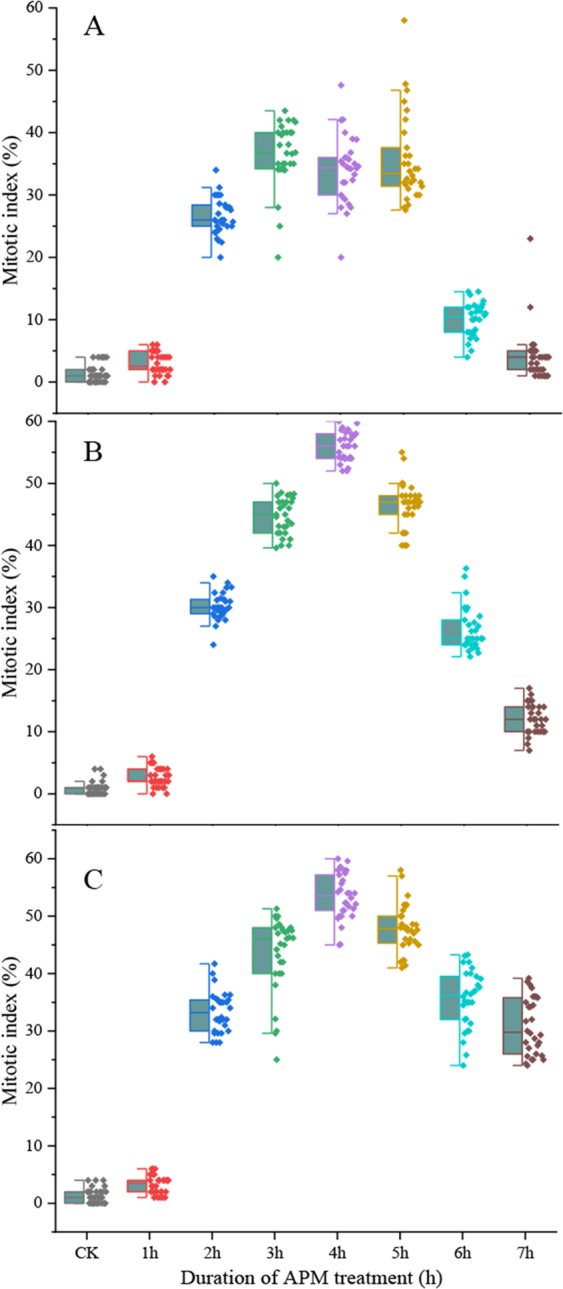
Metaphase index following treatment with different APM concentrations at 28 °C. (**A**) 2 μM, (**B**) 2.5 μM, (**C**) 3 μM. Thirty independent images with at least 3,000 nuclei per sample.

**Figure 8:**
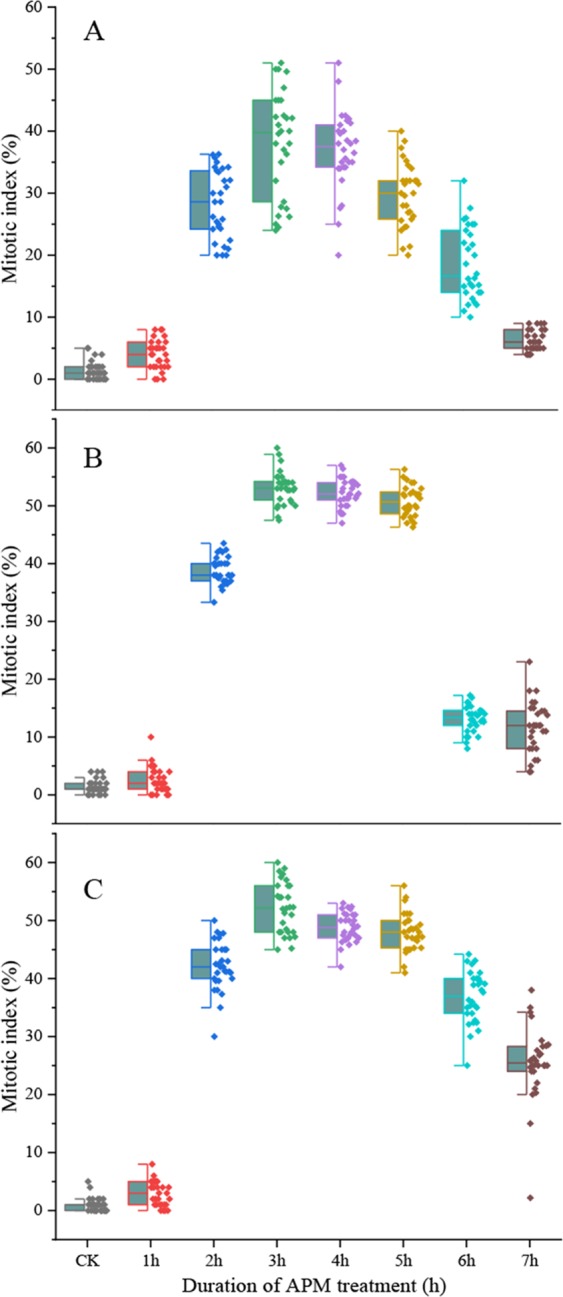
Metaphase index following treatment with different APM concentrations at 30 °C. (**A**) 2 μM, (**B**) 2.5 μM, (**C**) 3 μM. Thirty independent images with at least 3,000 nuclei per sample.

Based on the results for 25 °C, we also tested incubation in 2.0 μM, 2.5 μM or 3.0 μM APM solution at 28 °C and 30 °C. At 28 °C, the highest percentage of mitotic cells was 36.5%, 55.9% and 53.4% for incubation in 2.0 μM, 2.5 μM and 3.0 μM APM solution for 3 h, 4 h and 4 h, respectively (Fig. 7). After treatment with 2.5 μM APM, the proportion of mitotic cells increased sharply between 0–4 h after initiating treatment and peaked at 4 h. At 28 °C, cellular malformation was observed when the incubation time exceeded 3 h or the APM concentration was above 2.5 μM. Then, it was better to use 2.5 μM APM solution for 3 h at 28 °C. At 30 °C, the highest percentage of mitotic cells was 38%, 53.1% and 52.2% following a 3 h incubation with 2.0 μM, 2.5 μM and 3.0 μM, respectively (Fig. 8). Based on these collective results, incubation in 2.5 μM APM solution for 3 h at 30 °C produced the highest proportion of mitotic cells.

### Preliminary screening of CCS methods at different temperatures

At 25 °C, the highest mitotic index was about 50.5%, which was seen for the CCS method involving incubation in 2 mM HU for 18 h, 0.1X HS without HU for 3.5 h, and then 2.5 μM APM for 3.0 h (Supplementary Dataset Fig. S3). At 28 °C, the highest mitotic index was 44.8%, using 2 mM HU for 18 h, 0.1X HS without HU for 2.5 h, followed by 2.5 μM APM for 3.0 h (Supplementary Dataset Fig. S3). At 30 °C, the highest mitotic index obtained was 53.1% with a CCS method involving 2 mM HU for 18 h, 0.1X HS without HU for 1.5 h, and 2.5 μM APM for 3.0 h (Supplementary Dataset Fig. S3). In general, temperature substantially influenced recovery time, wherein higher incubation temperatures were associated with shorter recovery times.

### Application

**Figure 9:**
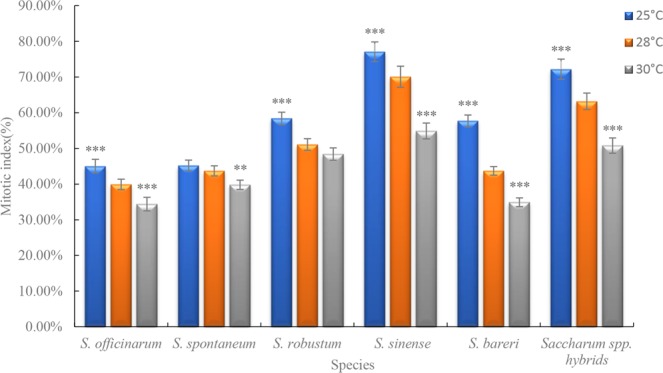
Average mitotic index for different species at different treatment temperatures. Thirty independent images with at least 3,000 nuclei per sample. The asterisks denote that it is on the level of significance in comparison to 28 °C in the same species (*stand for *p* < 0.05, **stand for *p* < 0.01 and ***stand for *p* < 0.001).

We next tested the CCS procedures at varying temperatures with different *saccharum* species to measure the stability and adaptability of the preliminary CCS screening results. The CCS procedures significantly increased the mitotic index of all species tested (Supplementary Dataset Fig. S4). The average mitotic index of *S. sinense* was higher than that for other species at different temperatures, whereas that for *S. officinarum* was the lowest (Fig. 9). Comparing results for different treatment temperatures for the same species, the average mitotic index decreased from 25 °C to 30 °C (Fig. 9), indicating that CCS parameters defined in the preliminary screening CCS method would be most effective at 25 °C compared to higher temperatures.

## Discussion

Sugarcane is an important crop for sugar and energy resources worldwide, but there are no reference genome sequences available due to the complexity of the sugarcane genome and high levels of polyploidy^4^. For genome studies of sugarcane, the ploidy can be reduced to single chromosome levels using flow cytometry, but a stable and efficient CCS protocol that allows enrichment of mitotic cells is needed.

Preparation of intact nuclear suspensions from sugarcane root tips is a prerequisite for flow cytometry. High quality nuclei suspensions have a clear background as well as low amounts of nuclei clumping and tissue fragments^12,13,22^. Thus, selection of a suitable lysis buffer is important to maintain nuclei stability in suspension and to protect against degradation of nuclear DNA^23^. In this study we showed that the G1, S and G_2_/M phase in nuclei suspensions prepared with LB01 and WPB buffers could be readily distinguished using flow cytometry. LB01 buffer is widely used to prepare nuclei and chromosome suspensions from plants including wheat, maize and mosquito grass^12,24,25^. Meanwhile, WPB buffer described by Loureiro *et al*. contains phenolic and mucilaginous compounds, which can complicate determination of DNA content using flow cytometry^23^. However, nuclei suspensions prepared using LB01 buffer produced wider G_2_/M peaks compared to those seen for WPB buffer, indicating that, for sugarcane, WPB buffer would be more suitable. This result could be explained by the presence of Na_2_S_2_O_5_ and PVP in WPB buffer that could combine with phenolic compounds in sugarcane roots.

For many plants, seeds are used as a starting material for CCS, but for sugarcane we instead used the cane stalk due to difficulties in the collection and cultivation of sugarcane seeds. The optimal concentration and treatment time for DNA synthesis inhibitors varies across plant species^9,19^. Treatment with HU solutions inhibits DNA synthesis causing cells to persist in the G_1_/S phase^16^. In previous studies, specimens from different plant species were treated for 18 h with solutions containing HU ranging from 1.0 mM to 4.5 mM^16,19,21,22,26^. Here, treatment of Badila roots with 2.0 mM HU solution for 18 h at 25 °C, 28 °C and 30 °C, resulted in 93.1%, 87.2% and 93.1%, respectively, of cells being in the G_1_/S phase. The optimal HU concentration and treatment times for Badila were also effective for *Avena sativa*, *Hordeum vulgare* and *Triticum aestivum*^27–29^. Meanwhile, no increase in the accumulation of G_1_/S phase cells was seen when the HU concentration was less than 2 mM, whereas treatment with 3 mM HU solution impeded cell cycle recovery in sugarcane.

The 0.1X HS recovery treatment allowed cells in the G_1_ and early S phase to proceed to the later S and G_2_ phases. The optimal recovery time for accumulation of S/G_2_ phase cells varied according to temperature and for Badila was 3.5 h, 2.5 h and 1.5 h at 25 °C, 28 °C and 30 °C, respectively. Cell cycle blockage at the G_2_/M phase can be achieved by disrupting the mitotic spindle through inhibition of microtubule polymerization^9,30^. Sensitive mechanisms regulate spindle movement by activating the bipolar spindle in cells such that each chromosome centromere aligns along the metaphase plate in the center of the cell^17,31^. Moreover, the tension at the centromere is relieved when spindle formation is inhibited and the cell cycle is halted, at which point CCS can occur^16,32^. Here we found that the mitotic index correlated with the concentration of the microtubule polymerization inhibitor APM and treatment time. APM concentrations below 2.5 μM did not markedly affect the accumulation of mitotic cells, yet when the APM concentration was too high or the treatment time too long, the mitotic cells that were produced were abnormal. Similar observations were made for common wheat and cotton^33,34^. For Badila, the optimal CCS protocol was incubation in 2.5 µM APM for 3 h at 25 °C, which produced a mitotic index of 50.5% (Fig. S3). Previous studies involving different plant species indicated that the maximum mitotic index could range from 40–80%^9,16,27,28,35^, which is within the range seen here for Badila (44–53%), and supports that the preliminary screening identified practical CCS methods for 25 °C, 28 °C and 30 °C treatment temperatures. Preliminary screening of CCS methods with main species of genus *Saccharum* at different temperatures showed that the average mitotic indices of *Saccharum* spp. hybrids and *S. sinense* were higher than that for other *Saccharum* species. Meanwhile, the average mitotic index of different sugarcane species was decreased with increasing incubation temperature. Thus, the CCS protocol using a 25 °C incubation temperature and a sequence of 2 mM HU for 18 h, 0.1X HS without HU for 3.5 h and 2.5 µM APM solution for 3.0 h, all at 25 °C, was the most stable and effective for sugarcane.

Despite originating from the same cane stalk, some roots remained asynchronous. To improve the mitotic index for sugarcane even further, several factors should be considered: i) the length of the roots used for processing should be as uniform as possible prior to initiating CCS; ii) the cane stalk, nuclei suspension buffer and other treatment solutions must be freshly prepared; and iii) the incubation temperature must be maintained at 25 °C. Moreover, since different species exhibited different mitotic indices, the recovery time can be modified to improve the index, particularly when chromosomes are sorted by flow cytometry. The recovery time at 25 °C can also vary across plants and species, as seen by the 2 h range for *Avena sativa*, *Triticum aestivum* and *Zea mays* (4.5 h, 5.5 h and 3.5 h, respectively)^24,27,29^. As such, extending the recovery period by 0.5–1.0 h according to sugarcane species could enhance enrichment in flow chromosome sorting, which is based on the metaphase index and chromosome length and shape.

## Conclusion

Compared with other lysis buffers, WPB buffer was optimal for preparing nuclei suspensions from sugarcane roots. Upon application of parameters identified in preliminary screening of CCS protocols using Badila to several main species of genus *Saccharum* at incubation temperatures of 25 °C, 28 °C and 30 °C, the average mitotic index was decreased as the temperature increased. As such, the optimal CCS protocol to obtain a mitotic index of at least 50% starting with sugarcane root tips was: 2 mM HU for 18 h, 0.1X Hoagland’s Solution without HU for 3.5 h, and 2.5 μM APM for 3.0 h at 25 °C.

## Supplementary information

Dataset 1.
