# Supplementary material for: A flow cytometry-based analysis to establish a cell cycle synchronization protocol for Saccharum spp
Source: Sci Rep. 2020 Mar 19;10:5016. doi: 10.1038/s41598-020-62086-9 (PMC7081271; doi:10.1038/s41598-020-62086-9)
Supplement: Supplementary file 1 — Dataset 1. [file 41598_2020_62086_MOESM1_ESM.pdf]

A flow cytometry-based analysis to establish a cell cycle synchronization protocol for *Saccharum* spp.

Shan Yang[1#], Kai Zeng[1#], Ling Luo[1], Wang Qian[1], Zhiqiang Wang[2], Jaroslav Doležel[3], Muqing Zhang[2], Xiangxiong Gao[4], Zuhu Deng[1, 2] *

[1]National Engineering Research Center for Sugarcane, Fujian Agriculture and Forestry University, Fuzhou, China, 350002

[2]State Key Laboratory for Protection and Utilization of Subtropical Agro-Bioresources, Guangxi University, Nanning, China, 530004

[3]Centre of Plant Structural and Functional Genomics of the Institute of Experimental Botany, Olomouc, Czech, CZ-78371

[4]College of Horticulture, Fujian Agriculture and Forestry University, Fuzhou, China, 350002

*__Correspondence__
Zuhu Deng
dengzuhu@163.com

[#]These authors contributed equally to this work.

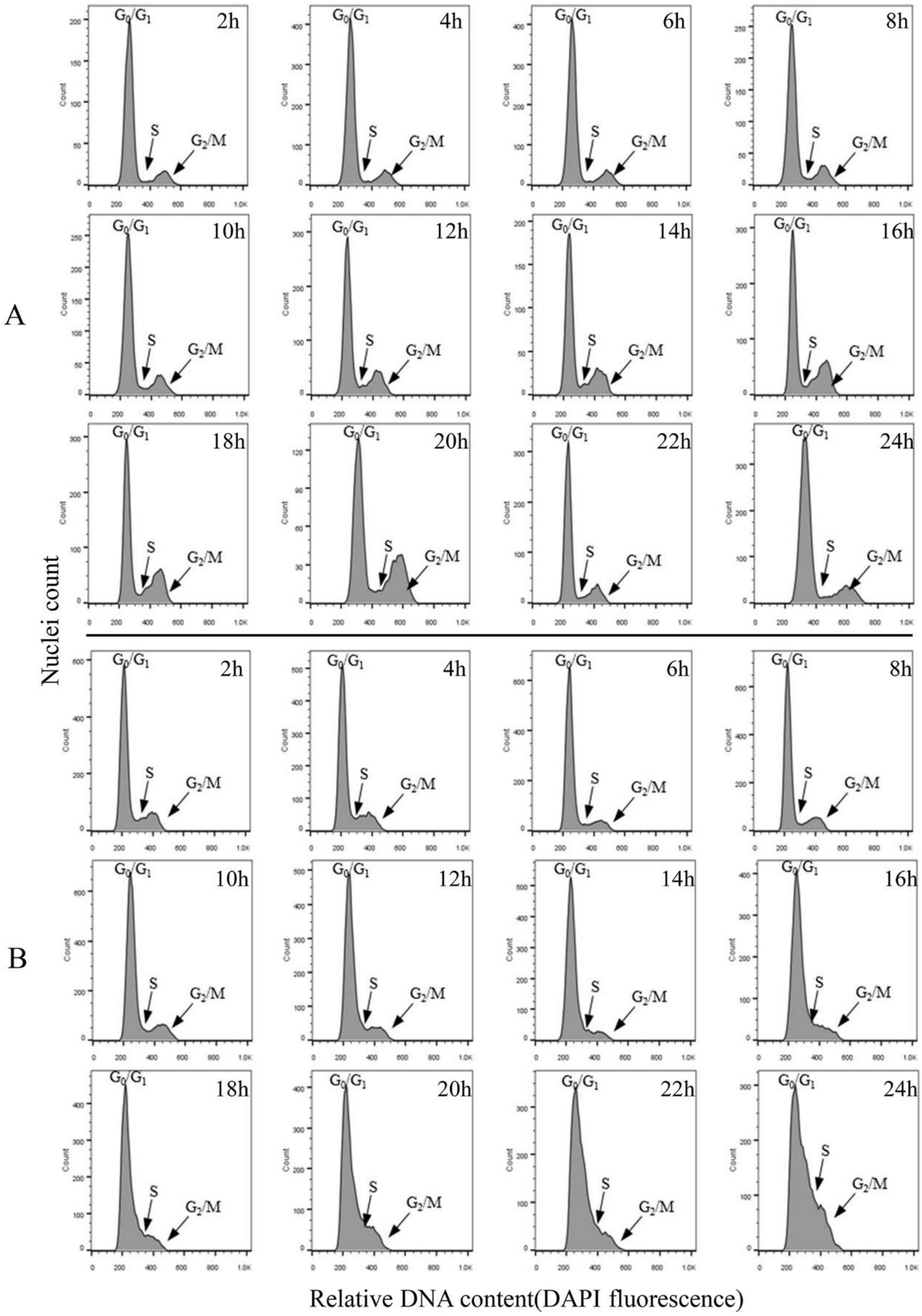

Figure S1. Flow cytometry results for different HU treatment times at 25 ℃. A: control treatment; B: 2 mM HU treatment.

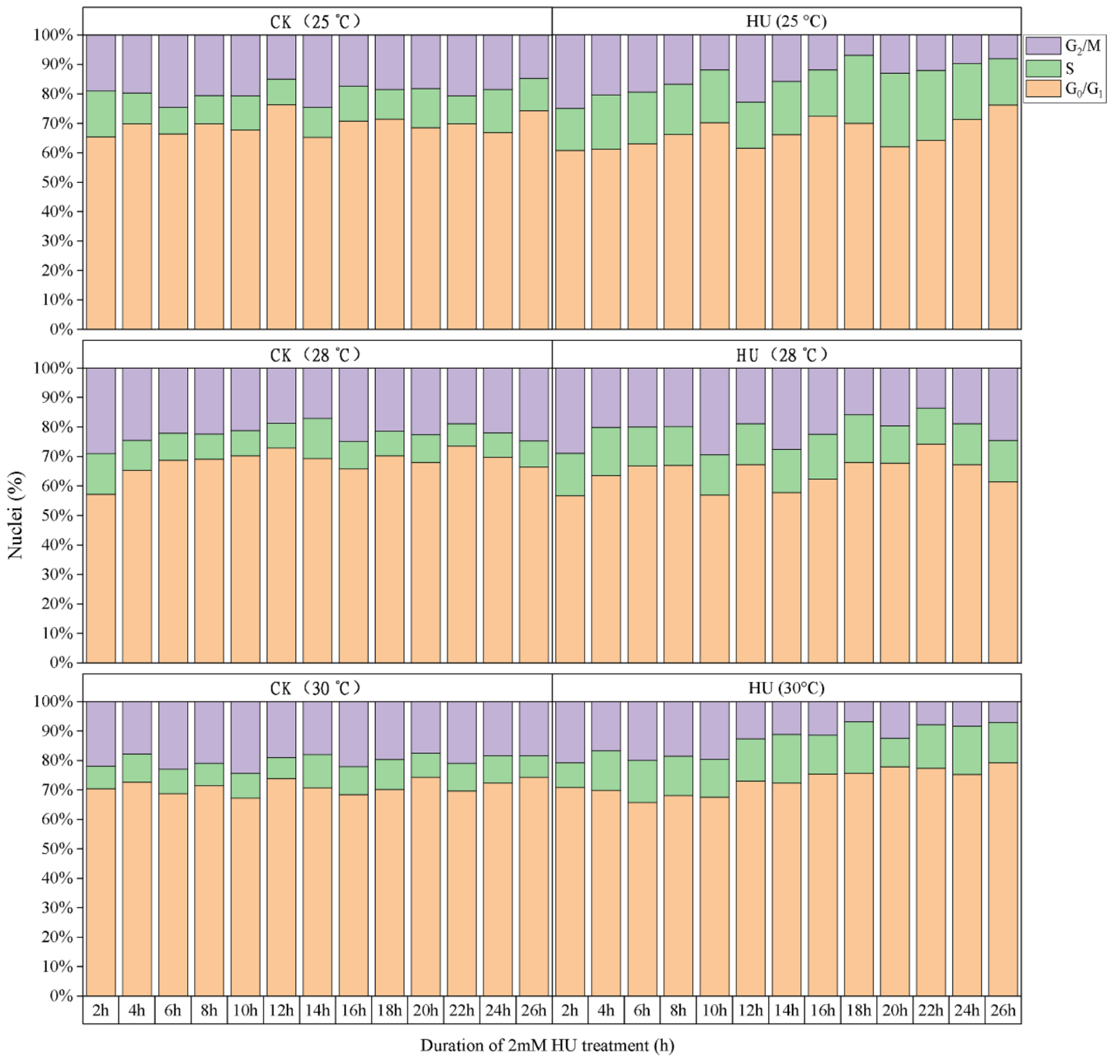

Figure S2. Percentage of cells in the G_0/G_1, S and G_2/M phase following treatment with 2 mM HU at different temperatures.

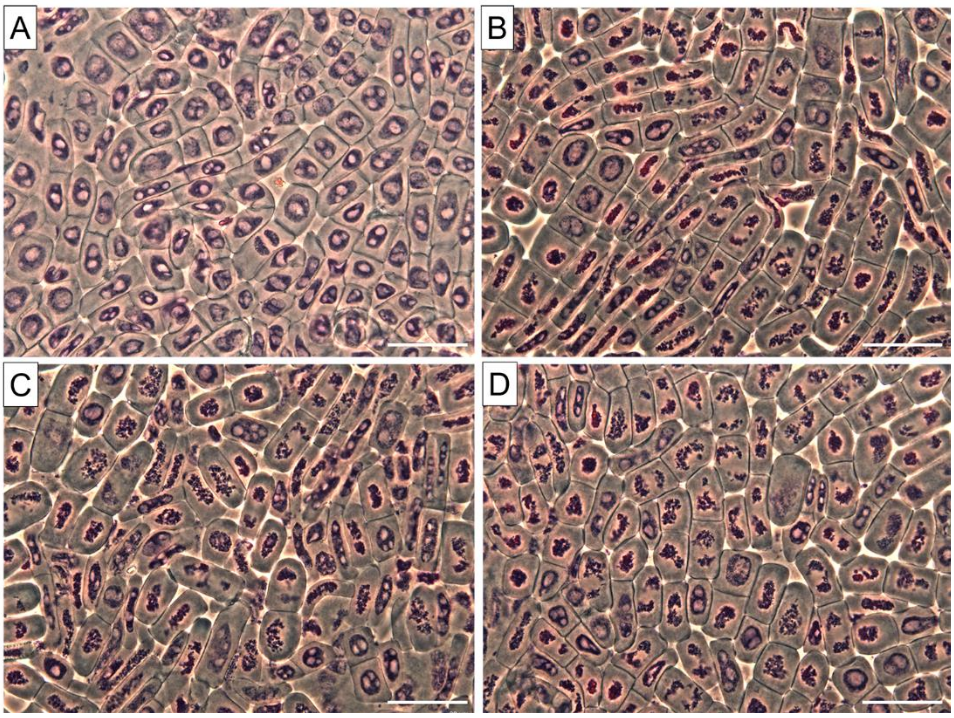

Figure S3. Badila root tip cells in preliminary screening of CCS treatment conditions. A: CK; B: 25 ℃; C: 28 ℃; D: 30 ℃. Scale bar = 50 μm.

Full size figures:

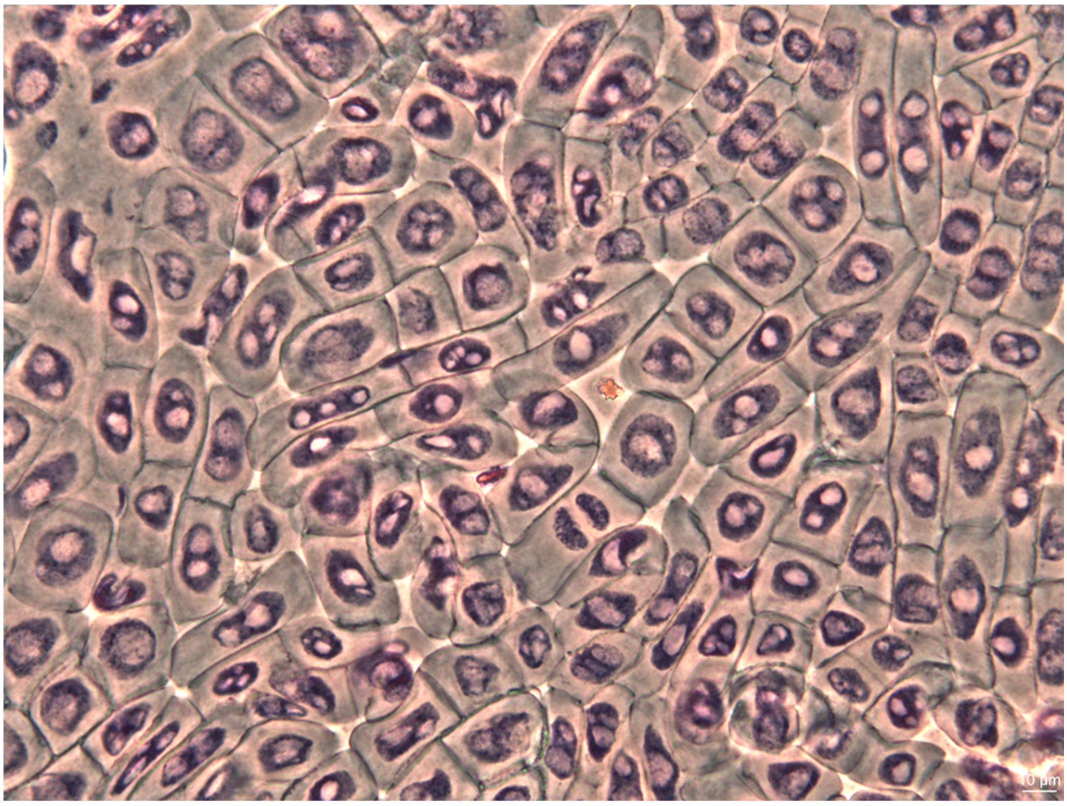

Figure S3-A Badila root tip cells without preliminary screening of CCS treatment conditions.

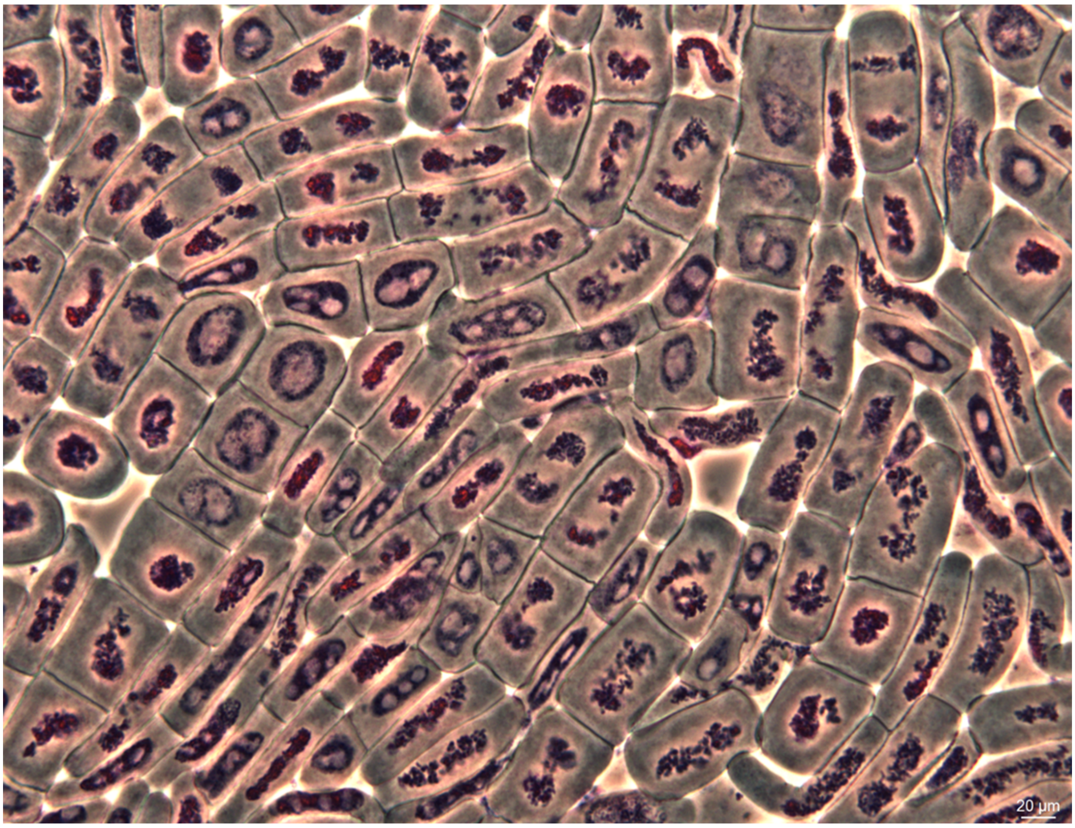

Figure S3-B. Badila root tip cells in preliminary screening of CCS treatment conditions at 25 ℃.

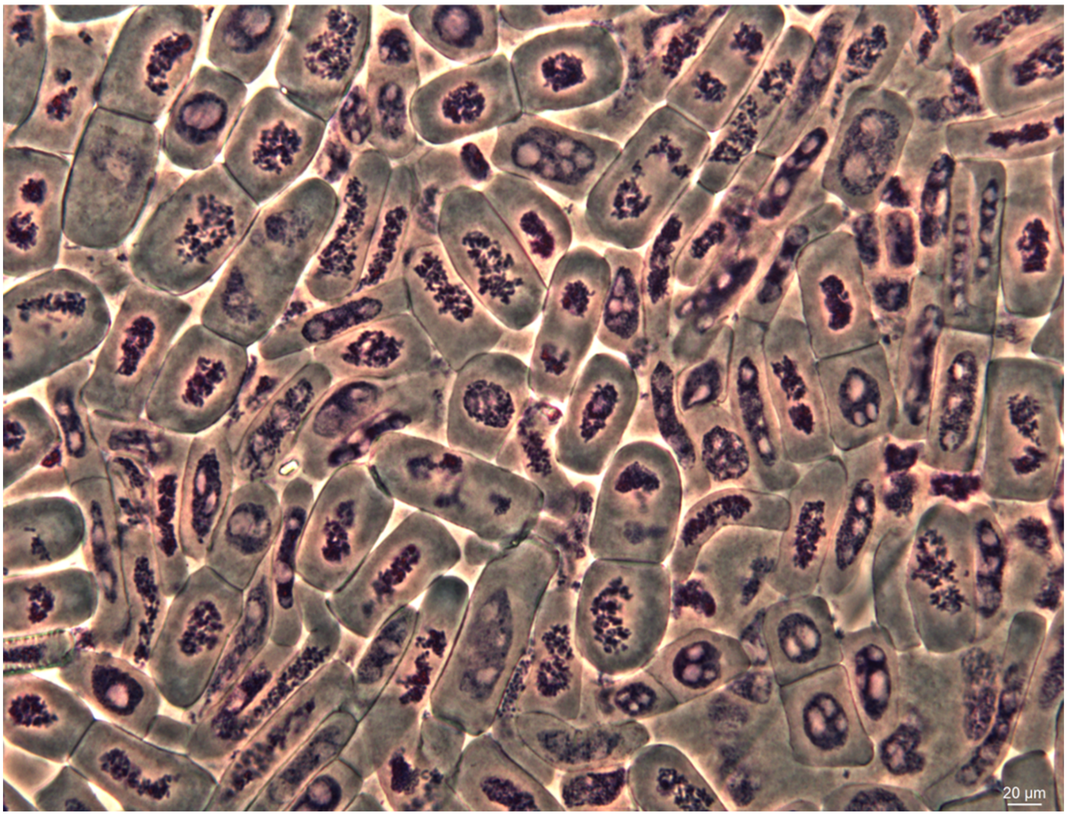

Figure S3-C. Badila root tip cells in preliminary screening of CCS treatment conditions at 28 ℃.

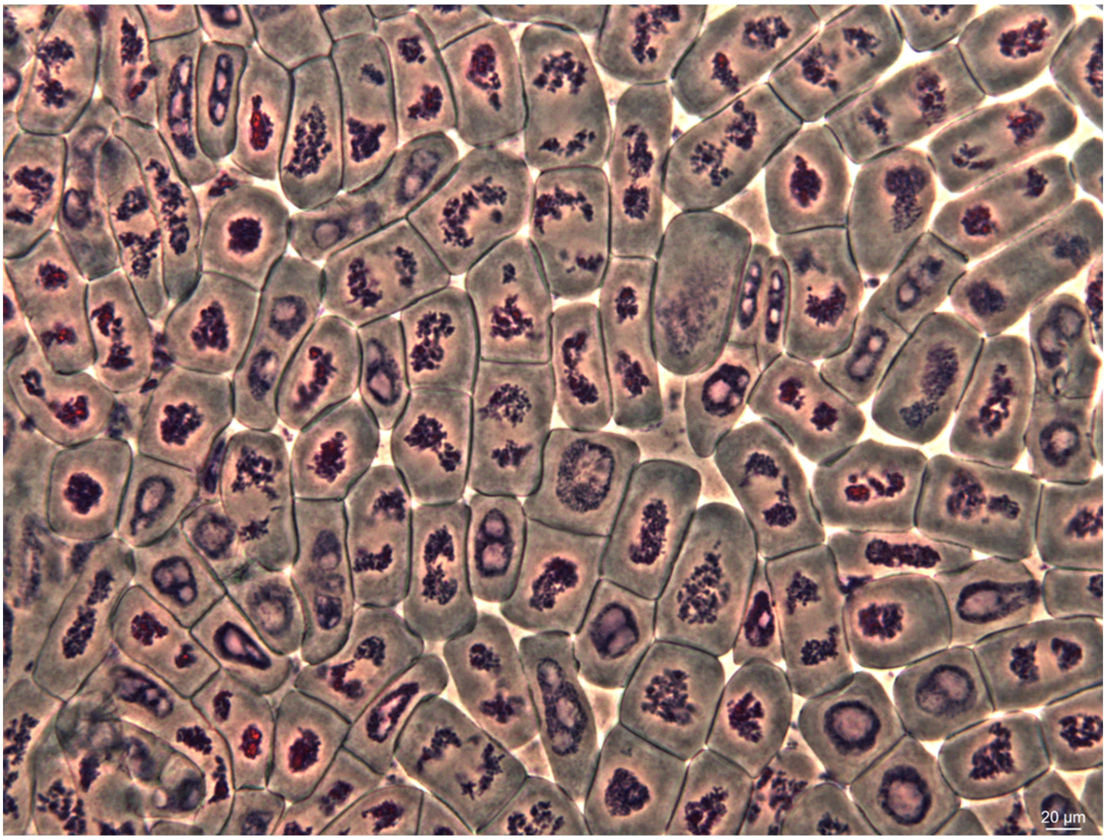

Figure S3-D. Badila root tip cells in preliminary screening of CCS treatment conditions at 30 ℃.

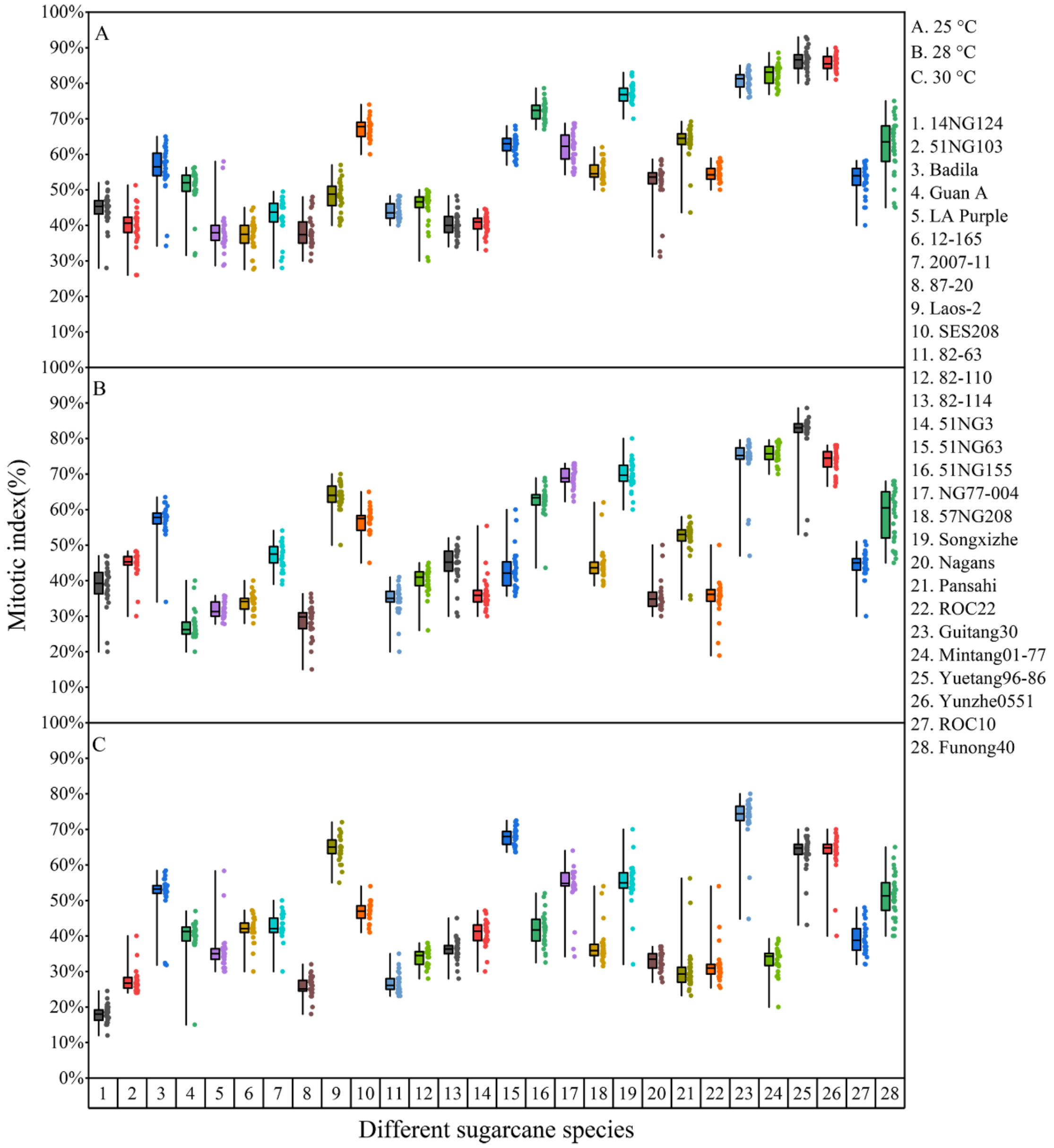

Figure S4. Mitotic index of different sugarcane species after synchronization treatment at different temperatures.
